# BOOST: Medical Image Steganography Using Nuclear Spin Generator

**DOI:** 10.3390/e22050501

**Published:** 2020-04-26

**Authors:** Bozhidar Stoyanov, Borislav Stoyanov

**Affiliations:** Konstantin Preslavsky University of Shumen, 9712 Shumen, Bulgaria

**Keywords:** steganography, nuclear spin generator, medical image, peak signal-to-noise ratio, key space calculation

## Abstract

In this study, we present a medical image stego hiding scheme using a nuclear spin generator system. Detailed theoretical and experimental analysis is provided on the proposed algorithm using histogram analysis, peak signal-to-noise ratio, key space calculation, and statistical package analysis. The provided results show good performance of the brand new medical image steganographic scheme.

## 1. Introduction

In this century, with the rapid evolution of data processing and information technologies, web security instruments are becoming more and more relevant. Various health systems are constantly relocating into the cloud and mobile device space. A body of US national rules for the defence of certain medical information must be taken into account for secure communication [1,2]. Many technologies have been introduced in recent years for secure storage and transmission of medical records and information regarding patient identity, such as digital watermarking [3,4], image encryption [5,6,7,8,9], and steganography [10,11].

Nevertheless, most of those schemes depend on some form of cryptography. The aim of cryptography is to create and analyze protocols that prevent individuals or the public from reading private data. In cryptography, an encryption is the method of encoding data. This method converts the original representation of the data, known as input text, into an alternative form known as encrypted text. Only authorized parties can decrypt encrypted data back to input text and access the original data [12]. Unlike cryptography, steganography is the art and science of hiding in plain sight secret data without being detected inside an innocent objects, called containers, so that it can be safely transmitted on a public channel of communication [13,14]. Containers may have the form of video streams, audio records, and digital images.

Image steganography refers to the hiding of user data in an image file [15]. Medical image steganographic schemes play a significant function in contemporary therapeutic procedures. The digital security of medical records and patient data both during communication and at the storage location must be ensured [16]. For medical images, sensitive patient information is embedded as header details defined in the Digital Imaging and Communications in Medicine (DICOM) standard in the image files [17] and should be removed before network transmission.

The efficiency of the steganography methods can be calculated by the three valuable specifications: security, capacity, and visual undetectability [18,19].

Numerous strategies are employed to conceal a variety of input data with respect to medical images. Because of the resistance of increasing statistical attacks, use of chaotic functions in steganography algorithms becomes more popular. Satish et al. [20] introduced Logistic map based spread spectrum image steganography. Jain and Lenka [19] used an asymmetric cryptographic system for secret information hiding in brain images. Jain and Kumar [21] presented a medical record steganography method based on Rivest–Shamir–Adleman cryptosystem and decision tree for data inclusion. Jain et al. [22] described an improved medical image steganographic methodology using a public key cryptosystem and linear feedback shift register (LFSR), and dynamically picked diagonal blocks. Ambika and Biradar [23] proposed a novel technique to hide data in medical images. The scheme uses two level discrete wavelet transformation with a pixel selection by Elephant Herding–Monarch Butterfly algorithm. By using 1D chaotic function, medical image stego algorithm is presented in [24].

The steganography techniques provide the necessary security and privacy in data transmission. In our humble opinion, the main contributions of our work can be summarized as follows:

- We present novel algorithm for pseudorandom byte output using nuclear spin generator (NSG), which has acceptable statistical properties.
- We apply the pseudorandom algorithm to a novel medical image steganography scheme.
- We examine the proposed method, and the data show that it has excellent peak signal-to-noise ratio, strong collision resistance, and desirable security properties that can withstand most common theoretical and statistical attacks.

In Section 2, we present a novel pseudorandom byte output method based on two nuclear spin generators. In Section 3, we introduce the novel medical image steganography algorithm BOOST and complete steganalysis is given. Finally, the article is concluded in Section 4.

## 2. Pseudorandom Byte Output Algorithm Using Nuclear Spin Generator

Pseudorandom generators are basic primitives used in cryptography algorithms but in our case we apply the random properties of pseudorandom byte generator to steganography algorithm. Pseudorandom generators are software realized methods for extracting sequences of random values.

### 2.1. Proposed Pseudorandom Byte Output Algorithm

**Figure 1:**
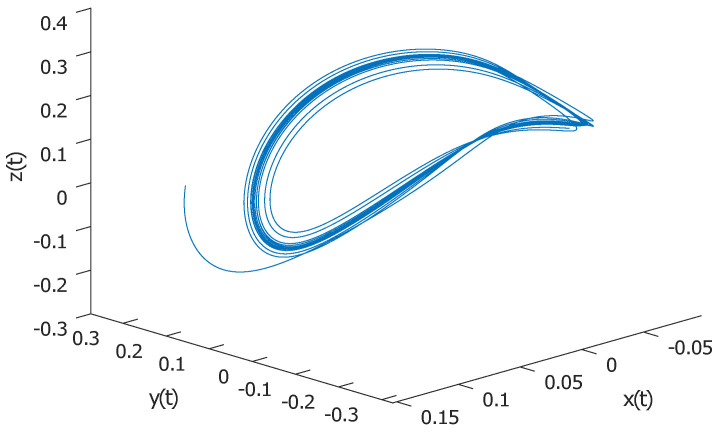
Nuclear spin generator in 3D phase space.

**Figure 2:**
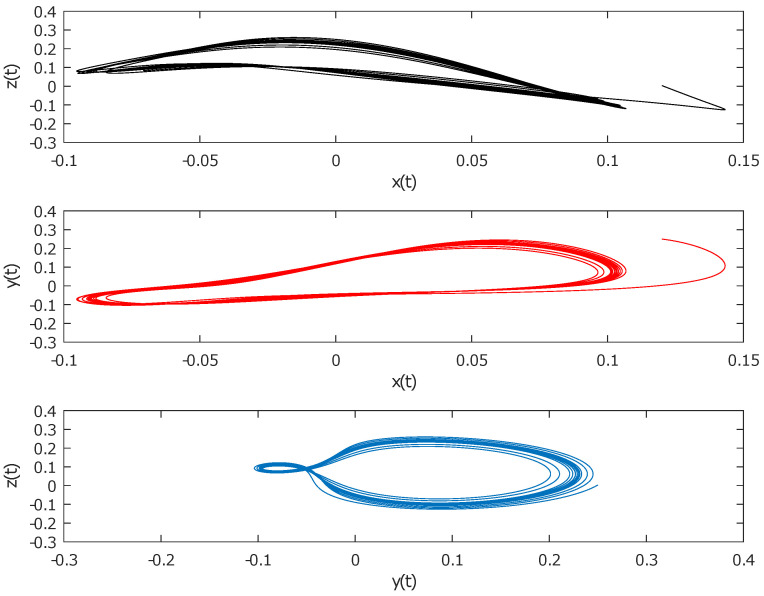
Nuclear spin generator time series.

The nuclear spin generator is a high-frequency oscillator which generates and controls the oscillations of the motion of a nuclear magnetization vector in a magnetic field. This system exhibits a large variety of regular and dynamic motions [25,26,27,28,29]. The nuclear spin generator was first described by Sherman [30]. The typical NSG is nonlinear three-dimensional dynamical system given by

(1) $$\begin{array}{cc} \dot{x}\left(t\right) & =-\beta x+y\\ \dot{y}\left(t\right) & =-x-\beta y(1-kz)\\ \dot{z}\left(t\right) & =\beta (\alpha \left(1-z\right)-ky^{2}), \end{array}$$

where *x*, *y*, and *z* are the components of the nuclear magnetization vector in the *X*, *Y*, and *Z* directions, respectively, and α, β, and *k* are positive parameters. The nuclear spin generator with initial values (x,y,z)=(0.12,0.25,0.0032) and parameters equal to (α,β,k)=(0.15,0.75,21.5) is plotted in Figure 1 and Figure 2.

The novel pseudorandom byte output algorithm is based on the next few steps:

1. The seed values x(0), y(0), and z(0) from Equation (1) are determined. The output byte length *L* is determined.
2. Equation (1) is iterated for *N* times.
3. The iteration of the nuclear spin generator continues. As a result, the three floating-point values x(i), y(i), and z(i) are calculated. They are manipulated as follows: $xm\left(i\right)=mod(abs\left(int\left(x\left(i\right)\times 10^{13}\right)\right)),256)$, $ym\left(i\right)=mod(abs\left(int\left(y\left(i\right)\times 10^{13}\right)\right)),256)$, and $zm\left(i\right)=mod(abs\left(int\left(z\left(i\right)\times 10^{13}\right)\right)),256)$, where abs(a) returns the modulus of *a*, int(a) returns the the integer part of *a*, truncating the value behind the decimal sign, and mod(a,b) returns the reminder after division.
4. Perform XOR operation between xmi, ymi, and zmi to get an output byte.
5. Return to Step 3 until the output byte length *L* is reached.

### 2.2. Key Size Analysis

The set of all initial values compose the key size. The key size of the proposed pseudorandom generator has three secret values x(0), y(0), and z(0). As reported by IEEE floating-point standard [31], the computational precision of the 64-bit double-precision number is about $10^{-14}$. The key size of the proposed scheme is ${\left(10^{14}\right)}^{3}=10^{42}\approx 2^{139}$ bits. This is high enough against mechanisms of exhaustive attack [32].

### 2.3. Statistical Tests

To estimate unpredictability of the novel nuclear spin equation based pseudo-random byte generator, we used National Institute of Standards and Technology (NIST) statistical software [33] and ENT [34] statistical application. Using the novel pseudorandom byte generator, 3000 sequences of 125,000 bytes were produced.

The NIST package contains 15 statistical tests: frequency, block frequency, cumulative sums forward and reverse, runs, longest run of ones, rank, spectral, non overlapping templates, overlapping templates, universal, approximate entropy, serial first and second, linear complexity, random excursion, and random excursion variant. The application calculates the proportion of streams that pass the particular tests. The range of acceptable proportion is determined using the confidence interval, defined as

$$\hat{p}\pm 3\sqrt{\frac{\hat{p}\left(1-\hat{p}\right)}{m}},$$

where $\hat{p}=1-\alpha$ and *m* is the number of binary tested sequences. NIST recommends that, for these tests, the user should have at least 1000 sequences of 1,000,000 bits each. In our setup, m=3000. Thus, the confidence interval is

$$0.99\pm 3\sqrt{\frac{0.99(0.01)}{3000}}=0.99\pm 0.0054498.$$

The proportion should lie above 0.9845502 with exception of random excursion and random excursion variant tests. These two tests only apply whenever the number of cycles in a sequence exceeds 500. Thus, the sample size and minimum pass rate are dynamically reduced taking into account the tested sequences.

The distribution of *p*-values is examined to ensure uniformity. The interval between 0 and 1 is divided into 10 subintervals. The *p*-values that lie within each subinterval are counted. Uniformity may also be specified through an application of a $\chi^{2}$ test and the determination of a *p*-value corresponding to the goodness-of-fit distributional test on the *p*-values obtained for an arbitrary statistical test, *p*-value of the *p*-values. This is implemented by calculating

$$\chi^{2}=\sum_{i=1}^{10}\frac{{\left(F_{i}-s/10\right)}^{2}}{s/10},$$

where $F_{i}$ is the number of *p*-values in subinterval *i* and *s* is the sample size. A *p*-value is computed such that *p*-$value_{T}=IGAMC\left(9/2,\chi^{2}/2\right)$, where IGAMC is the complemented incomplete gamma statistical function. If *p*-$value_{T}\geq 0.0001$, then the sequences can be considered to be uniformly distributed.

The output values of the first 13 test are in Table 1. The minimum pass rate for each statistical test with the exception of the random excursion variant test is approximately 2953 for a sample size of 3000 binary sequences. The random excursion test outputs eight *p*-values, which are tabulated in Table 2. The random excursion variant test outputs 18 randomness probability values: *p*-values, as shown in Table 3. The minimum pass rate for the random excursion variant test is approximately 1788 for a sample size of 1819 binary sequences.

The output results in Table 1, Table 2 and Table 3 indicate that all *p*-values are uniformly distributed over the (0,1) interval. The total numbers of acceptable streams are within the expected confidence levels for all performed tests. Based on the results, the novel pseudo-random byte generator passed without error NIST suite.

The ENT consists of six statistical tests (entropy, optimum compression, $\chi^{2}$ square, arithmetic mean value, Monte Carlo for π, and serial correlation), which focus on the pseudorandomness of byte sequences. We tested a stream of 375,000,000 bytes of the proposed generator. The value of entropy is 8.0 byte per byte; the optimum compression would reduce the byte file by 0%; $\chi^{2}$ square is 238.18 (randomly would exceed this value 76.79% of the times; the sequence is random); arithmetic mean value is 127.5040 (very close to 127.5, less then 10%); Monte Carlo for π is 3.141616448 (error 0.00%); and serial correlation coefficient is 0.000003 (less then 0.005 for true random generators). The novel pseudorandom byte generator passed successfully ENT tests.

Based on the excellent test outputs, we can infer that the proposed pseudorandom byte generator has satisfying statistical properties and provides reasonable level of security.

## 3. Medical Image Steganography Using Nuclear Spin Generator

### 3.1. Embedding Scheme

In this subsection, by using the pseudorandom byte generation algorithm based on the nuclear spin function in Section 2, we present a medical image steganography algorithm named BOOST.

We consider 16 bits DICOM grayscale input images of n×n size. As input message, we specify the patient information (text based patient medical records with patient identification data). The information includes patient name, patient ID/UID, and doctors remarks. Stego image is the input image with embedded encrypted patient information. The DICOM header data are directly transferred into stego image, based on [35].

The proposed medical image steganography algorithm BOOST consists of the following steps:

1. Iterate for *L* times the pseudorandom generator based on the nuclear spin generator in Section 2.
2. Apply XOR operation between the pseudorandom byte sequence and all of the input message to produce an encrypted bytes *C*.
3. Specify the input intervals of gray levels [a,b] of non-black pixels, where *a* and *b* determine the boundaries of the container.
4. Index the image pixels by consecutive passing through columns and separate those that fall within the interval [a,b].
5. Convert encrypted data to binary sequence using ASCII table.
6. Consecutively embed the encrypted data into the last bits of the pixels from the interval [a,b]
7. The list output pixels is checked to see if their new values are in the input interval. For those pixels that fall outside this range, their value increases by +2 if their new values are below the minimum value of the interval or decreases by −2 if the maximum value of the range is exceeded.

### 3.2. Extraction Scheme

1. Retrieve the number *L* of embedded bytes, input levels interval [a,b], and the secret key space of the pseudorandom generator based on the nuclear spin generator in Section 2.
2. Index the image pixels by consecutive passing through columns and separate those that fall within the interval [a,b].
3. Consecutively extract the embedded data from the last bits of the pixels from the interval [a,b].
4. Iterate for *L* times the pseudorandom generator based on the nuclear spin generator in Section 2.
5. Apply XOR operation between the output pseudorandom byte sequence and all of the extracted bytes to produce the input bytes *C*.

**Figure 3:**
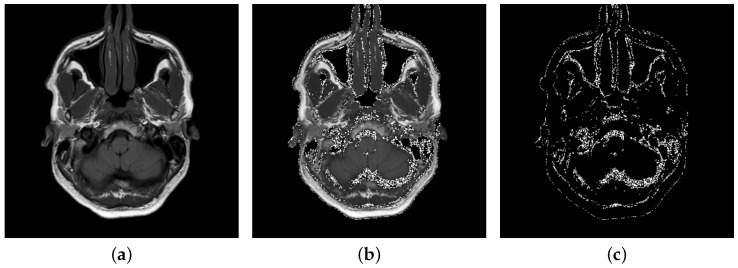
Illustration of embedding a message using the BOOST method and input levels interval [20,48]: (**a**) the original input image Brain IM_0001; and (**b**,**c**) the location of embedded message.

The proposed medical image steganography algorithm was implemented in C++ programming language. Fifteen 16-bit monochrome DICOM images were used for the experimental analysis. The test images were selected from the National Electrical Manufacturers Association (NEMA) medical image database: ftp://medical.nema.org/medical/dicom/DataSets/WG16/Philips/ClassicSingleFrame/. The folder consists of classical 16 bits DICOM grayscale single frame medical images of brains, knees, and livers. An example to illustrate the BOOST is presented in Figure 3.

### 3.3. Steganographic Analysis

**Figure 4:**
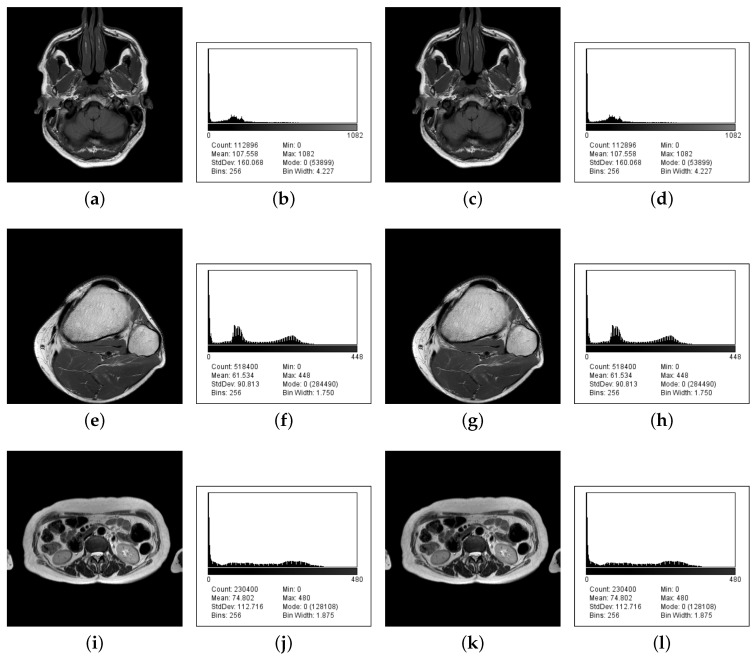
(**a**,**e**,**i**) Input images Brain IM_0001, Knee IM_0001, and Liver IM_0001; (**b**,**f**,**j**) their histograms; (**c**,**g**,**k**) stego images; and (**d**,**h**,**l**) their histograms.

An image histogram is an accurate illustration of the tonal value distribution in digital images. This check compares both input and stego image histograms. Histograms, performed using ImageJ2x 2.1.5.0 (http://www.rawak.de/rs2012/), for three input images and their stego images are also shown in Figure 4.

It is considered that the histograms of the stego images are much the same as those of the input images with no evidence of hidden messages in stego images.

Peak Signal-to-Noise Ratio (PSNR) is the proportion between the highest possible value of a signal and the value of distorting noise that affects the accuracy of its representation. It is defined as:

(2) $$PSNR=10\log_{10}\frac{{\left(2^{d}-1\right)}^{2}}{MSE}\left(dB\right),$$

where *d* is the bit depth of the pixel and MSE is the Mean-Square Error between the input and stego images. MSE is defined as:

(3) $$MSE=\frac{1}{mn}\sum_{i=1}^{m}\sum_{j=1}^{n}{\left(P\left[i,j\right]-S\left[i,j\right]\right)}^{2},$$

where P[i,j] and S[i,j] are the *i*th row and *j*th column pixel in the input and stego images, respectively.

In Table 4, we provide the computed values for MSE and PSNR for BOOST algorithm. MSE and PSNR are calculated for images with 1050 bytes (8400 bits), 1042 bytes (8336 bits), and 1119 bytes (8952 bits) embedded. Maximum payload is calculated as a number of non-black pixels.

From results obtained, as shown in Table 4, the PSNR values are extremely high, above 113 dB, which suggests an excellent level of security for the proposed BOOST algorithm.

The Bit Error Rate (BER) is computed as the actual number of bit positions which are changed in the stego image compared with the input image. A value of BER close to 0.0 stands for high efficiency of the steganography algorithm. The Normalized Cross-Correlation (NCC) calculates the cross-correlation in the the frequency domain, depending on the size of the images. Then, it computes the local sums by pre-computing running sums. Use local sums to normalize the cross-correlation to get correlation coefficients. The output matrix holds the correlation coefficients, which can range between −1.0 and 1.0. NCC is defined as:

(4) $$NCC=\frac{\sum_{i=1}^{m}\sum_{j=1}^{n}\left(P\left[i,j\right]\times S\left[i,j\right]\right)}{\sum_{i=1}^{m}\sum_{j=1}^{n}{\left(P\left[i,j\right]\right)}^{2}}.$$

A value of NCC close to 1.0 represents perfect quality of the stego image.

The Structural SIMilarity (SSIM) index is an algorithm for measuring the similarity between input and stego images [36]. The output SSIM index is a decimal number between −1 and 1. Value 1 indicates excellent structural similarity.

In Table 5, we provide the calculated values for BER, NCC, and SSIM for the presented BOOST scheme. From the obtained results shown in Table 5, it is clear that the BER are very close to 0.0 and NCC and SSIM values are almost equal to 1.0. The data indicate that the BOOST scheme provides good quality and excellent structural similarity.

The resistance of the BOOST algorithm against cropping attack [37,38] was tested. Cropping is the mechanism by which outer parts of the image are cut. Three stego images (Brain IM_0001, Knee IM_0001, and Liver IM_0001) generated from the BOOST algorithm were subjected to cropping attacks.

The normalized correlation (NC) values were calculated for the stego image and the corresponding cropped image [38]. The output NC results varied between 0.8944 and 1, as shown in Table 6. We see from these results that the proposed BOOST algorithm reasonably resists cropping attack.

The steganographic analysis undoubtedly shows the good rate of the proposed algorithm. Table 7 summarizes some of the computed values of our proposed scheme with other algorithms.

Using the given test results, we can conclude that the presented algorithm BOOST, based on the nuclear spin generator, has satisfying statistical properties and provides a proper safety expectation.

## 4. Conclusions

We introduce a novel medical image steganographic scheme named BOOST. The presented algorithm uses a novel pseudorandom byte output technique based on the nuclear spin generator. Our security investigation (mean square error, peak signal-to-noise ratio, normalized cross-correlation, and structural similarity) shows that the proposed hiding can be used with success for secure medical record communication.

## Figures and Tables

**Table 1:** National Institute of Standards and Technology (NIST) test suite results.

NIST Test	*p*-Value	Pass Rate	Results
Frequency	0.633649	2972/3000	Success
Block frequency	0.014996	2964/3000	Success
Cumulative sums forward	0.928857	2976/3000	Success
Cumulative sums reverse	0.053059	2977/3000	Success
Runs	0.215195	2970/3000	Success
Longest run of ones	0.158133	2974/3000	Success
Rank	0.851939	2971/3000	Success
Spectral	0.552383	2955/3000	Success
Non overlapping templates	0.489210	2970/3000	Success
Overlapping templates	0.117661	2967/3000	Success
Universal	0.800626	2971/3000	Success
Approximate entropy	0.092411	2971/3000	Success
Serial first	0.646836	2963/3000	Success
Serial second	0.410055	2970/3000	Success
Linear complexity	0.370821	2974/3000	Success

**Table 2:** NIST Random excursion test results.

State	*p*-Value	Pass Rate	Result
−4	0.042839	1793/1819	Success
−3	0.176043	1792/1819	Success
−2	0.958805	1800/1819	Success
−1	0.821611	1791/1819	Success
+1	0.905874	1801/1819	Success
+2	0.932163	1804/1819	Success
+3	0.395583	1798/1819	Success
+4	0.695564	1793/1819	Success

**Table 3:** NIST Random excursion variant test results.

State	*p*-Value	Pass Rate	Result
−9	0.136979	1804/1819	Success
−8	0.218022	1805/1819	Success
−7	0.458964	1806/1819	Success
−6	0.250128	1805/1819	Success
−5	0.368209	1805/1819	Success
−4	0.210521	1806/1819	Success
−3	0.821611	1805/1819	Success
−2	0.365446	1800/1819	Success
−1	0.475836	1796/1819	Success
+1	0.927657	1804/1819	Success
+2	0.183647	1805/1819	Success
+3	0.457919	1799/1819	Success
+4	0.188110	1795/1819	Success
+5	0.286462	1798/1819	Success
+6	0.750377	1794/1819	Success
+7	0.957844	1793/1819	Success
+8	0.916782	1794/1819	Success
+9	0.542519	1798/1819	Success

**Table 4:** Mean-Square Error(MSE) and Peak Signal-to-Noise Ratio (PSNR) results.

Input Image	Image Size	Maximum Payload	Percent Volume	Available Levels	Input Levels	Message (Bytes)	MSE	PSNR (dB)
Brain IM_0001	336 × 336	83,179	73.68	1083	[50,146]	1050	0.0191	113.5238
Brain IM_0002	336 × 336	83,362	73.84	851	[50,146]	1050	0.0192	113.4977
Brain IM_0003	336 × 336	83,557	74.01	823	[50,146]	1050	0.0191	113.5218
Brain IM_0004	336 × 336	83,341	73.82	875	[50,146]	1050	0.0190	113.5319
Brain IM_0005	336 × 336	83,883	74.30	834	[50,146]	1050	0.0191	113.5198
Knee IM_0001	720 × 720	249,148	48.06	449	[30,56]	1042	0.0041	120.1618
Knee IM_0002	720 × 720	250,531	48.33	426	[30,56]	1042	0.0043	120.0302
Knee IM_0003	720 × 720	251,867	48.59	461	[30,56]	1042	0.0043	120.0263
Knee IM_0004	720 × 720	256,834	48.54	453	[30,56]	1042	0.0042	120.0637
Knee IM_0005	720 × 720	260,969	50.34	444	[30,56]	1042	0.0042	120.0558
Liver IM_0001	480 × 480	109,631	47.58	481	[20,68]	1119	0.0098	116.4055
Liver IM_0002	480 × 480	112,992	49.04	581	[20,68]	1119	0.0100	116.3465
Liver IM_0003	480 × 480	114,107	49.53	626	[20,68]	1119	0.0103	116.2160
Liver IM_0004	480 × 480	115,670	50.20	643	[20,68]	1119	0.0098	116.4325
Liver IM_0005	480 × 480	116,373	50.51	624	[20,68]	1119	0.0098	116.4383

**Table 5:** Bit Error Rate (BER), Normalized Cross-Correlation (NCC), and SSIM (Structural SIMilarity) results.

Image	BER	NCC	SSIM
Brain IM_0001	0.0012	0.9999971	0.9999787
Brain IM_0002	0.0012	0.9999950	0.9999757
Brain IM_0003	0.0012	0.9999934	0.9999838
Brain IM_0004	0.0012	0.9999968	0.9999769
Brain IM_0005	0.0012	0.9999955	0.9999809
Knee IM_0001	0.00026	0.9999979	0.9999806
Knee IM_0002	0.00027	0.9999982	0.9999794
Knee IM_0003	0.00027	0.9999979	0.9999720
Knee IM_0004	0.00027	0.9999980	0.9999682
Knee IM_0005	0.00026	0.9999976	0.9999581
Liver IM_0001	0.00061	0.9999982	0.9998838
Liver IM_0002	0.00062	0.9999973	0.9998954
Liver IM_0003	0.00064	0.9999970	0.9999311
Liver IM_0004	0.00061	0.9999983	0.9999308
Liver IM_0005	0.00061	0.9999984	0.9999253

**Table 6:** Normalized correlation (NC) results against cropping attack.

Cropping Attack		Brain IM_0001	Knee IM_0001	Liver IM_0001
Percent	10%	0.999	0.9872	0.9858
	20%	0.981	0.9729	0.9724
	30%	0.8944	0.9455	0.9093

**Table 7:** Comparison of our medical image steganography with other techniques.

Algorithm	Minimum Calculated PSNR (dB)	Capacity Bits per Pixel	Maximum Calculated BER
Proposed	113.50	0.74	0.0012
[16] Mantos 2016	103.68	0.5	-
[37] Thiyagarajan 2013	74.36	-	0.004
[22] Jain 2017 Improved	72.17	0.37	-
[39] Elhoseny 2018	57.02	-	0.0
